# Extracellular vesicles in urine of women with but not without kidney stones manifest patterns similar to men: a case control study

**DOI:** 10.1186/s13293-015-0021-2

**Published:** 2015-02-24

**Authors:** Muthuvel Jayachandran, Ghiara Lugo, Hillary Heiling, Virginia M Miller, Andrew D Rule, John C Lieske

**Affiliations:** Department of Surgery, College of Medicine, Mayo Clinic, 200 First Street SW, Rochester, MN 55905 USA; Department of Physiology & Biomedical Engineering, College of Medicine, Mayo Clinic, 200 First Street SW, Rochester, MN 55905 USA; Department of Internal Medicine, Division of Nephrology and Hypertension, College of Medicine, Mayo Clinic, 200 First Street SW, Rochester, MN 55905 USA; Laboratory Medicine and Pathology, College of Medicine, Mayo Clinic, 200 First Street SW, Rochester, MN 55905 USA

**Keywords:** Microvesicles, Microparticles, Exosomes, Urinary vesicles, Sex differences, Vesicles, Flow cytometry

## Abstract

**Background:**

The lifetime incidence of kidney stones is about two times greater in men compared to women. Extracellular vesicles (EVs) shed from activated cells are present in the urine and may reflect or even mediate renal physiology and/or pathology. This study was designed to standardize methodology to characterize urinary EVs by digital flow cytometry and to identify possible sex differences in EVs in persons with and without their first symptomatic kidney stones.

**Methods:**

Twenty-four-hour urine collections were obtained from persons presenting with their first kidney stone episode (*n* = 50 women, 60 men; age 19–76 years) and sex- and age-matched controls from the general population (*n* = 24 women, 36 men).

**Results:**

*Standardization*: Size of EV was variable within all groups. EV positivity was verified with two fluorophores for surface phosphatidylserine and/or using two different protein markers specific for renal-specific cells. The number of phosphatidylserine- and exosome marker-positive EVs did not correlate with urine osmolality and were similar in fresh vs. frozen and between two sequential urine collections from the same individual. *Sex differences*: Urine from women controls contained greater (*P* < 0.05) numbers of EVs positive for phosphatidylserine, exosomes, inflammatory factors and adhesion molecules, and cell-specific markers from different segments of the nephron, renal pelvis, and bladder compared to control men. In contrast, urine from women with kidney stones contained significantly (*P* < 0.05) lower numbers of EVs derived from podocytes, parietal cells, proximal convoluted tubule, thin and thick loop of Henle, distal tubule, collecting duct, renal pelvis, and bladder compared to control women and contained similar quantities of these types of EVs in men with and without kidney stones. There were also no sex differences in EVs positive for cell adhesion (E-cadherin and inter-cellular adhesion molecule-1 [ICAM-1]) molecules.

**Conclusions:**

Unlike women who do not have kidney stones, EVs in urine from women with nephrolithiasis are similar to men with and without kidney stones. Thus, EVs may mediate or reflect aspects of kidney stone pathogenesis and perhaps provide clues regarding sex differences in kidney stone incidence rates.

## Background

Approximately 10–12% of the general population is afflicted with at least one kidney stone in their lifetime [1], and the incidence appears to be rising in the USA and Europe [2,3]. The lifetime incidence of kidney stone disease is greater in men (12%) compared to women (5%), although kidney stone incidence rises in women after menopause [4,5]. Multiple factors contribute to kidney stone risk including age, sex, diet, geography, and genetic factors [2,6-8].

The majority (70–80%) of stones are composed of calcium oxalate (CaOx), often admixed with calcium phosphate. Changes in urine composition resulting in supersaturation of the urine are associated with a higher risk of developing a CaOx kidney stone. However, the exact series of events that connect super-saturation to formation of a kidney stone remain unclear. Thus, these stones are often referred to as “idiopathic” calcium oxalate stones. Furthermore, men are more likely to have a calcium oxalate or uric acid stone, while women are more likely to have a hydroxyapatite or struvite stone [9]. The cellular and molecular factors contributing to these sex differences in kidney stone risk are incompletely understood. Some studies support a role for renal tubular cell injury in the pathophysiology of nephrolithiasis [10-12]. For example, renal cells are injured by increased urinary concentrations of lithogenic molecules (e.g., oxalate) or calcium-containing crystals [12]. Injured and activated cells shed distinct populations of biologically active extracellular vesicles (EVs) that are involved in numerous pathophysiological processes by removal and exchange of excess and unwanted RNAs, proteins, receptors, and metabolites. Recent studies also suggest that activated cell-derived EVs can help identify early and late pathophysiological processes and contribute to the diagnosis, prognostic assessment, and management of individuals with suspected renal diseases [13,14]. However, a detailed profile of cellular injury or markers of cellular activation that could elucidate potential mechanisms involved in stone formation has yet to be developed.

**Figure 1:**
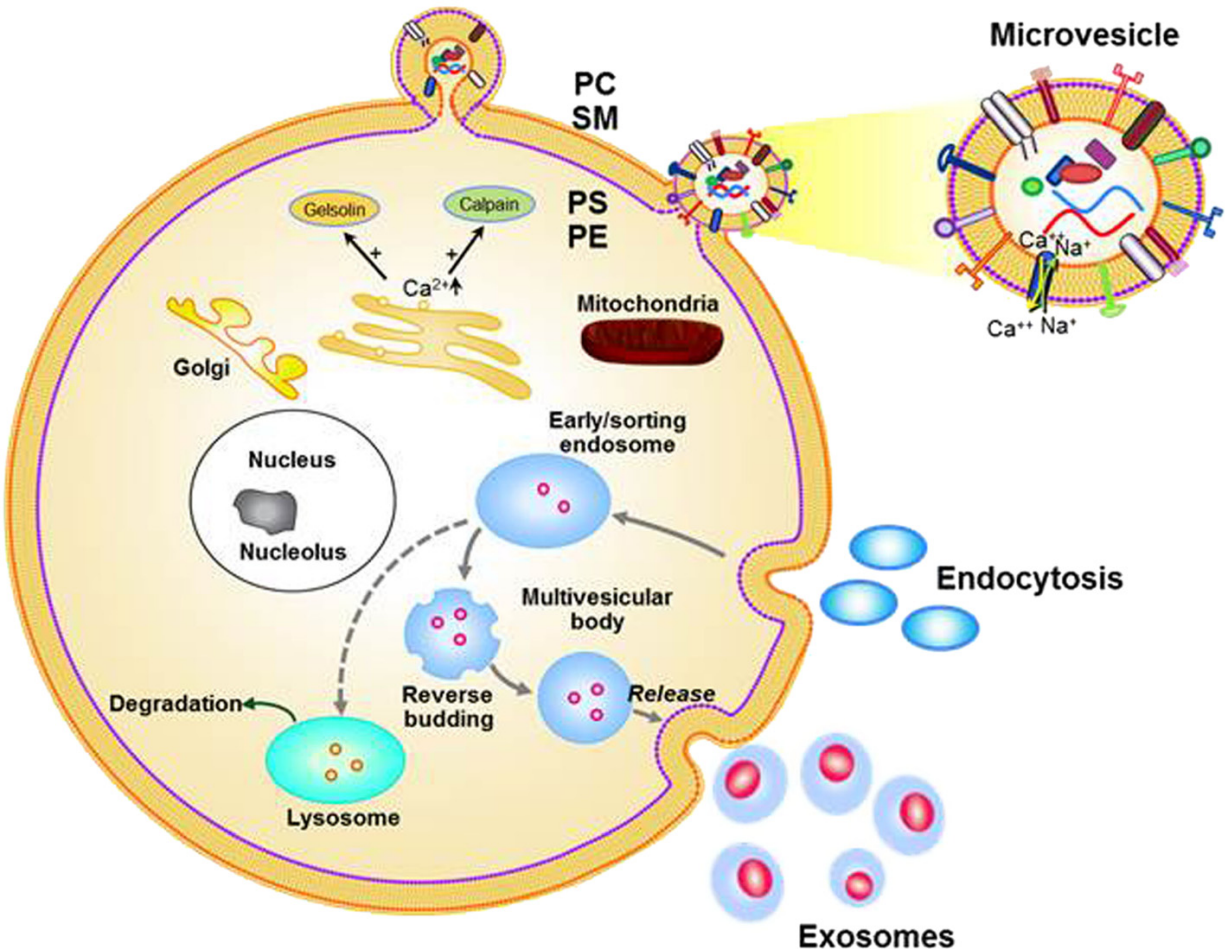
**Biogenesis of EVs (~0.1–1 μm in size; microvesicles from plasma membrane and exosomes (~30–100 nm) from multivesicular body) from cells during physiological and pathological condition.** Abbreviations: *PC* phosphatidylcholine, *SM* sphingomyelin, *PS* phosphatidylserine, *PE* phosphatidylethanolamine.

Therefore, the present study was designed to quantify and characterize the cellular origin of EVs (Figure 1) in a 24-h urine from first time kidney stone formers and age-matched persons without a history of kidney stones in the general population of Olmsted County, MN. Our hypotheses were as follows: (1) that the number and types of urinary EVs would differ between men and women and between persons with and without kidney stones and (2) that the nature of these differences may help to design larger and/or molecular studies to understand the sex differences in kidney stone disease. Complete characterization of urinary EVs in kidney stone formers may lead to identify novel cellular processes associated with kidney stone formation and potential tools for risk stratification and identification of early kidney stone or other renal disease processes, holding the potential to develop personalized treatment strategies based on the sex of the person.

## Methods

### Chemicals, reagents, and antibodies

Recombinant annexin-V and mouse anti-human CD9, CD54, CD63, and CD106 antibodies conjugated with fluorescein isothiocyanate (FITC) or R-phycoerythrin (PE) and TruCOUNT™ (4.2 μm) beads were purchased from BD Biosciences, San Jose, CA. Fluorescent latex beads (1 and 2 μm) were purchased from Sigma-Aldrich, Saint Louis, MO. Fluoresbrite® microparticles (0.2, 0.5, 1, and 2 μm) were purchased from Polysciences, Inc., Warrington, PA. FITC-conjugated mouse anti-human tissue factor was purchased from American Diagnostica Inc., Stamford, CT. Mouse anti-human podocalyxin-Alexa Fluor® 488 was purchased from R&D Systems, Inc., Minneapolis, MN. FITC-conjugated rabbit anti-TSG (tumor susceptibility gene) 101, anti-nephrin, anti-aquaporin-1, anti-aquaporin-2, and anti-cytokeratin 17 (CK17) antibodies were purchased from Biorbyt, Cambridge, UK. Rabbit anti-NPHS/Podocin, anti-synaptopodin, anti-claudin-1, anti-galectin, anti-cytokeratin 8, ant-Lrp (low-density lipoprotein-related protein 2)/megalin, anti-OAT4L/URAT1, anti-SLC12A3/NKCC (Na-K-Cl cotransporters), anti-prominin 2, anti-ATPVOD2/V-ATPase (vacuolar-type H^+^-ATPase), anti-CK19, anti-CK20, and anti-E-cadherin/CD234 polyclonal antibodies conjugated with FITC or PE were purchased from Bioss, Boston, MA. PE-conjugated mouse anti-human epidermal growth factor receptor (EGFR) and anti-human monocyte chemotactic protein-1 (MCP-1) antibodies were purchased from BioLegend Inc., San Diego, CA. FITC-conjugated mouse anti-human uromodulin were purchased from LSBio LifeSpan BioSciences, Inc., Seattle, WA. Mouse anti-human CD10 (neprilysin) was purchased from eBiosciences, Inc., San Diego, CA. 4-(2-Hydroxyethyl)-1-piperazineethanesulfonic acid (HEPES) and Hanks’ balanced salts were purchased from Sigma Chemicals Co., St. Louis, MO. All other reagents and solvents used in this study were of analytical/reagent grade.

### Study participants

This study was approved by the Institutional Review Board at Mayo Clinic, Rochester, MN. All participants gave written informed consent. Urine from randomly selected study participants of the Mayo Clinic O’Brien Urology Research Center Olmsted County population of symptomatic first time kidney stone formers and age-/sex-matched (19–76 years old) men and women without a history of kidney stones was used in this study. Participants completed study visits that included a structured questionnaire to obtain demographics, past medical history, and medical comorbidities.

### Urine sample collection and preparation for this study

Two 24-h urine samples 3 months apart were collected on a free-choice diet using mailed collection materials, written instructions, and verbal instruction provided by a study coordinator. Aliquots of 24-h urine samples were centrifuged at 3,400 rpm for 10 min to remove cells and larger molecular weight protein aggregates. Fresh or frozen (−80°C) cell-free urine samples were used in this study.

### Electron microscopy

**Figure 2:**
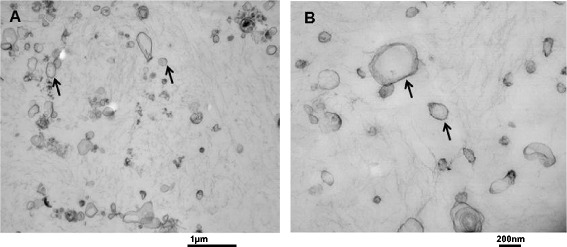
**Representative transmission electron microscopy of urinary EVs isolated by high-speed centrifugation.** Arrow heads indicate membranes; lower **(A)** and higher **(B)** magnification of heterogeneous population of urinary EVs.

Fresh or frozen 24-h cell-free urine samples were centrifuged at 20,000 *g* for 30 min to pellet urinary EVs. After centrifugation, vesicle-free urine was either discarded or stored at −80°C for other analyses. Pelleted urinary EVs were washed with HEPES/Hank’s (H/H) buffer pH 7.4 by vortexing for 1–2 min and then centrifuged at the same speed for 30 min. This supernatant was discarded, and urinary EVs were examined by transmission electron microscopic analysis as previously described for peripheral blood microvesicle analysis [15]. The heterogeneous nature of EV size was confirmed by transmission electron microscopy (Figure 2).

### Urine biochemistry

All urine biochemistry measurements in the 24-h urine were performed in the Mayo Clinic Renal Testing Laboratory, Rochester, MN, using standard protocols.

### Characterization of urinary EVs by flow cytometer

**Figure 3:**
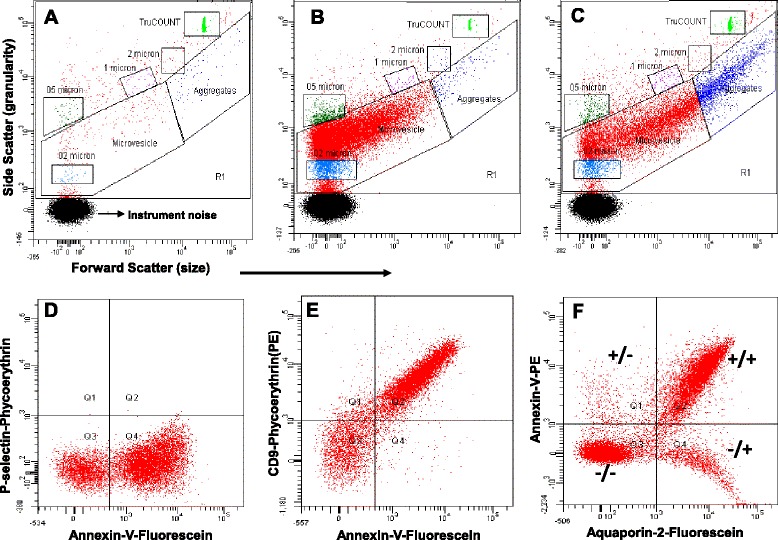
**Representative typical scatter plots (A–C) and fluorescence dot (quadrants) plots (D–F) from FACSCanto™** **flow cytometry, respectively. (A)** Control gates of buffer with fluorophore-conjugated antibodies and calibration beads. **(B)** Gates derived from diluted urine plus appropriate fluorophore-conjugated antibodies and calibration beads from one individual. **(C)** Gates derived from diluted urine plus appropriate fluorophore-conjugated antibodies and calibration beads from another individual. **(D)** Fluorescent dot plot (quadrants derived from microvesicle gate of Figure 3B) showing fluorophore spectra separate of annexin-V and P-selectin (activated platelet specific protein, irrelevant antibody control used for urine EV analysis). A similar pattern was observed with isotype control (fluorophore conjugated IgG) antibodies in diluted urine samples (data not shown). **(E)** Fluorescent dot plot (quadrants derived from microvesicle gate of Figure 3B) showing fluorophore spectra separate of annexin-V and CD9 antibody. **(F)** Fluorescent dot plot (quadrants derived from microvesicle gate of Figure 3B) showing fluorophore spectra separate of annexin-V and aquaporin-2 (a specific marker for collecting duct cell-derived EVs) fluorophore-positive (+) and fluorophore-negative (−) EVs. Similar fluorescent dot plots were obtained from diluted urine stained with specific antibodies for cells of the urinary tract (Figure 4, data not shown).

**Figure 4:**
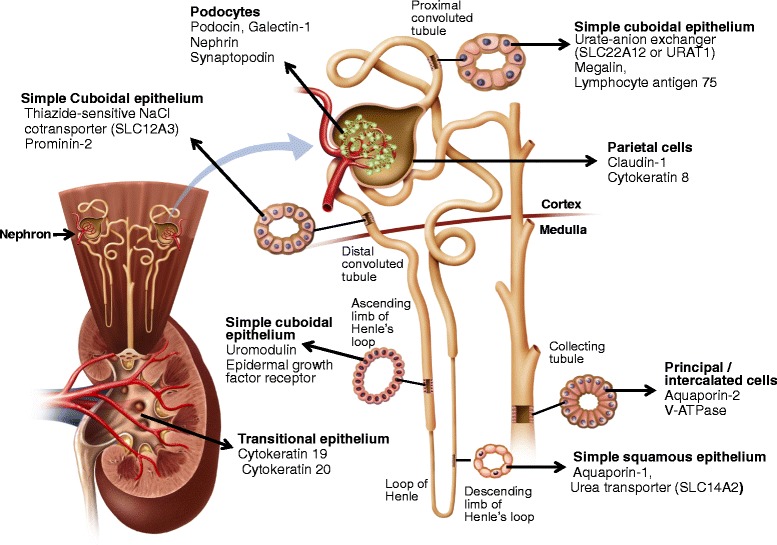
**Specific markers were used to identify EVs derived from cells of different segments of the nephron and renal pelvis.** All indicated markers were used in this study but only data of EVs positive for one or two markers of each cell type were presented in this manuscript.

Digital flow cytometry (FACSCanto™) was used to define EVs by size and annexin-V fluorescence. Gates to define the size of EVs were set using an internal standard of 0.2, 0.5, 1, and 2 μm Fluoresbrite® microparticles. Samples were spiked with a known quantity of 4.2μm diameter TruCOUNT™ (BD Biosciences, San Jose, CA) beads for quantification (Figure 3A–C). All buffers and antibodies were filtered twice through a 0.2 μm-sized membrane filter to eliminate chemical particles and to reduce instrument noise [15,16]. Acquisition gates for EVs with and without annexin-V or renal cell-specific monoclonal/polyclonal antibodies conjugated with fluorophore are shown in the microvesicle gate (Figure 3D–F). All the settings of flow cytometry were similar to those previously described for peripheral blood microvesicle analysis [15,16]. Urinary EVs were defined as events higher than 0.2 μm and less than 1 μm in diameter that were positive for annexin-V or cell-specific nephron markers (Figure 4). A considerable number of EVs greater than 1 μm in diameter were also observed in certain stone former and control urine samples. A 2 μm-sized upper limit for microvesicles was adopted as is standard for the analysis of peripheral blood [17]. To optimize the concentration and flow rate of urinary EVs for flow cytometric analysis, urine samples (20 μl) diluted with 80 μl of filtered H/H buffer pH 7.4 were incubated with 3–5 μl of annexin-V-fluorescein (FITC) for 30 min. After incubation, 800 μl of H/H buffer pH 7.4 plus 100 μl containing a defined quantity of TruCOUNT™ beads (BD Biosciences, San Jose, CA) was added to each sample tube prior to analysis by flow cytometer. The flow rate (low, medium, or high) was determined based on the threshold rate of events/second from the sample tube. For example, threshold events higher than 1,000 per second reduced the flow rate to low or medium and always maintained flow rate medium or low with less than 1,000 events/second. Very few samples had low threshold events less than 100 events/second, and therefore, the flow rate was increased to high in only these few samples. Gain settings of the flow cytometer were adjusted to place the TruCOUNT™ beads (4.2 μm) in the upper log for scatter. Unfiltered Isoton® II diluent from Beckman Coulter, Fullerton, CA, was used in the cytometer. Compensation for channel spill was calculated using the auto-compensation feature from recorded values of separate and combined unstained and single antibody-stained urinary EVs. Auto-calculated compensation parameters were verified weekly. Urinary EVs are defined in this study as events less than 1 μm (microvesicles) gate and higher than 1 μm in diameter gate and positive for annexin-V and cell-specific markers of nephrons (Figures 3 and 4). The thresholds were set with isotype controls or irrelevant fluorescent antibodies (Figure 3D). Urinary EVs stained in phosphate-buffered saline or HEPES-buffered saline (HBS; pH 7.4) without calcium served as negative controls for annexin-V positivity. The absolute count of urinary EVs either in the absence or presence of single or dual fluorescent staining was calculated with the following previously established method of calculation for blood microvesicles [15,16]. The volume of urine (ranging between 5 and 80 μl) was adjusted in the subsequent experiments in order to have at least 2,500 EVs in a microvesicle gate that stained positive with annexin-V, since it binds to phosphatidylserine expressed on the surface of EVs and allows their distinction from chemical particles in the urine. The absolute number of urinary EVs was expressed as the number of urinary EVs per microliter of urine and also normalized to urine creatinine concentration.

### Standardization and validation of urinary EV analysis

For standardization of urinary EV analysis, cell-free 24-h fresh and/or frozen urine samples from a mixed population (controls plus first time kidney stone formers) were used.

### Flow cytometry

**Figure 5:**
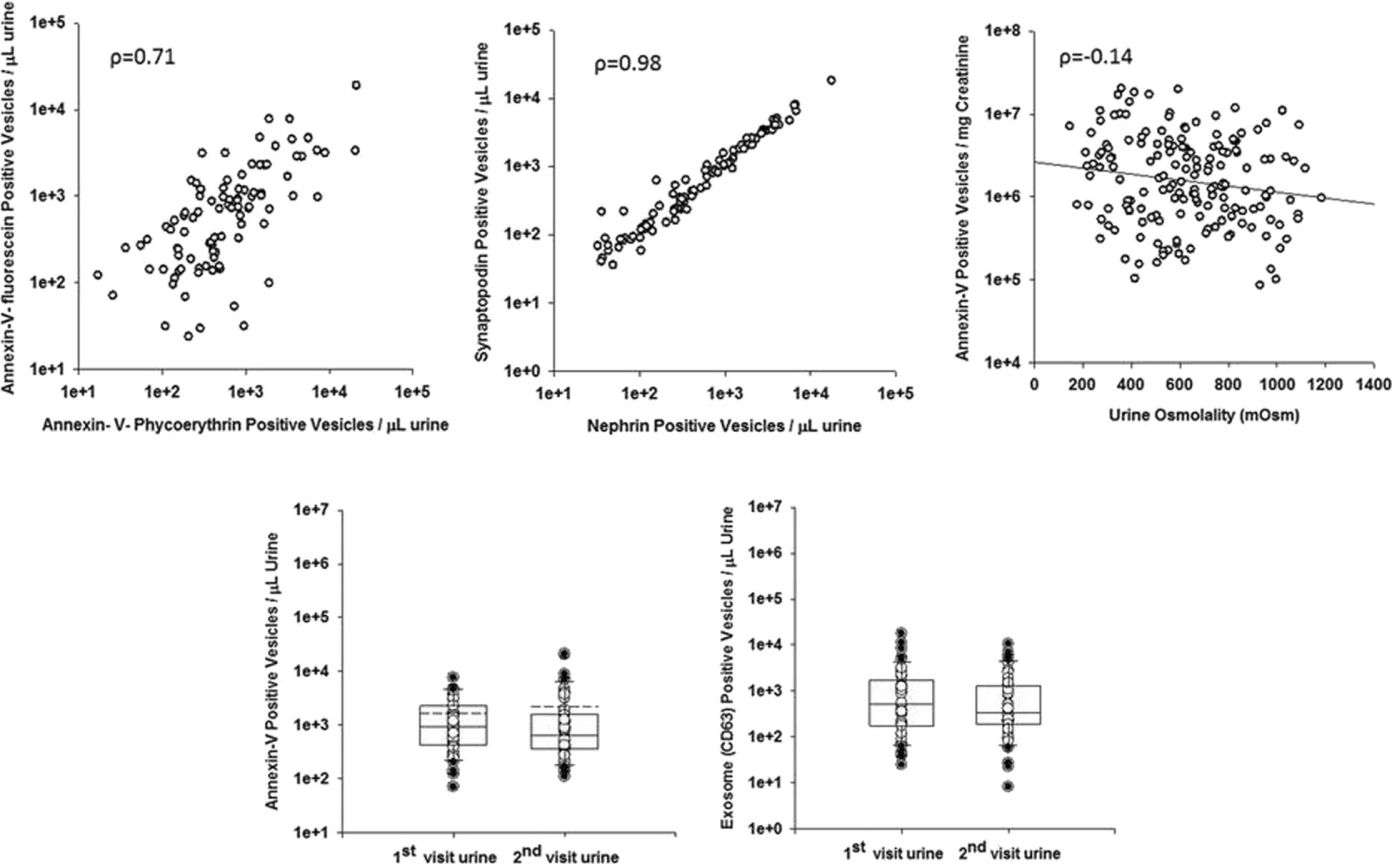
**Podocyte markers displaying high intra-individual correlation.** The upper left is the correlation of two different fluorophores conjugated to annexin-V and binding to surface phosphatidylserine on EVs from the same urine sample. The upper middle is the correlation of two distinct antibodies for the same cell-specific antigens (e.g., podocytes) binding to urinary EVs from the same urine sample. The upper right shows that annexin-V-positive vesicles did not correlate with urine osmolality. There were also no differences in the concentration of annexin-V- (lower left) and exosome marker (CD63, lower right)-positive EVs between two different 24-h urine collections obtained within 3 months from the same individuals.

Inter-individual variability of light scattering (size) was observed. For example, in one participant, greater than 95% of urinary EVs were less than 1 μm in size (microvesicle gate, Figure 3B), but in another, only 60–70% of EVs fell in the microvesicle gate with the remainder greater than 1 μm in size (aggregate gate, Figure 3C). This light-scattering heterogeneity was present across all sex and stone-forming groups. Therefore, all urinary EV data are presented for both microvesicle (<1 μm in size) and aggregate (>1 μm in size) gates. Annexin-V positivity was verified in 48 urine samples using two fluorophores FITC and PE that revealed a strong correlation (*ρ* = 0.71, Figure 5). Quantification of EV origin was verified in each case using two different protein markers specific for that nephron segment. Figure 4 depicts nephron segment-specific markers used to identify the source of urinary EVs. For example, podocyte-derived urinary EVs were verified by both nephrin and synaptopodin staining. Both of these podocyte markers displayed high intra-individual correlation (*ρ* = 0.98, Figure 5). The number of annexin-V-positive urinary EVs did not correlate with urine osmolality (*ρ* = −0.14, Figure 5) suggesting they were independent of urine flow rate. There was no difference in the concentration of urinary EVs positive for annexin-V and CD63 in the 24-h urine collection from the same person when visits 1 and 2 were compared (Figure 5). Further, the concentration of annexin-V-positive EVs measured in fresh versus frozen (few weeks to several months) urine collection of the same individual did not vary significantly (data not shown).

### Data analysis

Spearman’s correlation was used for comparisons of two fluorophores and two different protein markers for initial standardization. Clinical characteristics of study participants are presented as mean ± standard deviation (SD). Clinical characteristics, blood, and urine biochemistry data were analyzed by analysis of variance (ANOVA) followed by Student’s *t* test/Wilcoxon/Kruskal-Wallis (rank sums) test using JMP software and significance accepted at *P* < 0.05. Annexin-V- and exosome marker-positive EVs of nephron-derived urinary EVs are presented in total and by nephron/urinary tract segments in Tables 1 and 2 with median, 25th, and 75th percentiles. Differences in concentration of EVs between control women and men and kidney stone formers were analyzed by ANOVA followed by Mann-Whitney rank sum test using Sigma plot and/or Student’s *t* test/Wilcoxon/Kruskal-Wallis (rank sums) test using JMP software with significance accepted at *P* < 0.05.

**Table 1:** **Total number of phosphatidylserine, exosome, and inflammatory markers positive for urinary EVs**

	**Controls from the general population**		**First time kidney stone formers**	
	**Women ** **(** ***n*** **= 24)**	**Men ** **(** ***n*** **= 26–36)**	**Women ** **(** ***n*** **= 24–50)**	**Men ** **(** ***n*** **= 26–60)**
Urinary EV/mg creatinine				
Total phosphatidylserine positive	3,181 (1,144, 6,072)	697* (243, 1,158)	3,068 (1,143, 6,166)	1,416^†^ (515, 3,435)
Total CD63 (exosome) positive	911 (528, 1,258)	185* (86, 580)	2,308^†^ (525, 5,637)	1,062*^†^ (267, 2,628)
Cellular adhesion/inflammatory molecules positive for urinary EV/mg creatinine				
Total E-cadherin positive	6 (2, 21)	5 (2, 9)	7 (2, 17)	4 (2, 9)
Total ICAM-1 positive	184 (106, 320)	160 (33, 657)	204 (93, 627)	163 (43, 627)
Total VCAM-1 positive	720 (133, 1,708)	48* (6, 215)	46^†^ (15, 198)	35 (6, 206)
Total tissue factor positive	494 (214, 1,012)	204* (67, 667)	611 (241, 2,063)	421 (115, 1,962)
Total MCP-1 positive	20 (5, 36)	3* (2, 6)	8 (3, 16)	3* (1, 8)

Data are presented as median (25th, 75th percentile) × 10^3^/mg creatinine and analyzed by ANOVA followed by Student’s *t* test/Wilcoxon/Kruskal-Wallis (rank sums) test. *P < 0.05 between men and women of the same group; ^†^ *P* < 0.05 between control and stone formers of the same sex.

**Table 2:** **Numbers of urinary EVs from epithelial cells of different segments of nephron and urinary tract**

**Urinary EV/mg creatinine**		**Controls from general population**		**First time kidney stone formers**	
**Nephron segments**	**Markers**	**Women ** **(** ***n*** **= 24)**	**Men ** **(** ***n*** **= 26)**	**Women ** **(** ***n*** **= 24)**	**Men ** **(** ***n*** **= 26)**
Glomerulus	Podocin + galectin-1 positive	2,708 (1,037, 4,059)	24* (6, 183)	225^†^ (77, 292)	20* (7, 91)
Bowman’s capsule	Claudin-1 positive	96 (51, 322)	11* (8, 26)	11^†^ (4, 17)	14 (6, 35)
Proximal tubule	URAT1 positive	367 (195, 1,223)	39* (18, 61)	36^†^ (20, 74)	30 (21, 50)
Thin loop of Henle	SLC14A2 positive	182 (76, 544)	5* (3, 23)	11^†^ (6, 18)	9 (4, 50)
Thick loop of Henle	Uromodulin positive	21,960 (7,423, 46,782)	1,033* (236, 3,389)	2,652^†^ (482, 5,031)	1,458 (302, 4,558)
Distal tubule	SLC12A3 positive	611 (93, 1,459)	28* (14, 48)	24^†^ (8, 50)	15 (7, 42)
Collecting duct	V-ATPase positive	57 (11, 220)	16* (7, 23)	12^†^ (5, 46)	11 (4, 30)
Renal pelvis	Cytokeratin 19 positive	99 (51, 225)	21* (2, 37)	16^†^ (9, 25)	9 (6, 16)
Urinary bladder	Neprilysin positive	2,320 (1,308, 3,170)	222* (112, 427)	243^†^ (121, 382)	252 (126, 593)

Data are presented as median (25th, 75th percentile) × 10^3^/mg creatinine and analyzed by ANOVA followed by Student’s *t* test/Wilcoxon/Kruskal-Wallis (rank sums) test. *P < 0.05 between men and women of the same group; ^†^ *P* < 0.05 between control and stone formers of the same sex.

## Results

### Clinical characteristics

In general, the groups were comparable. Men with kidney stones were slightly older than the other three groups and tended to have a larger body mass index (Table 3). Overall, serum and urine chemistries were similar among groups, except for higher serum creatinine values in men (as expected).

**Table 3:** **Clinical characteristics, blood, and urine biochemistry of study participants**

	**Controls from the general population**		**First time kidney stone formers**	
	**Women (** ***n*** **= 24)**	**Men (** ***n*** **= 36)**	**Women (** ***n*** **= 50)**	**Men (** ***n*** **= 60)**
Clinical characteristics				
Age (years)	44 ± 12	42 ± 12	41 ± 16	51 ± 14*^†^
Body mass index (kg/m^2^)	28 ± 7 (*n* = 22)	28 ± 5 (*n* = 32)	28 ± 8 (*n* = 33)	31 ± 7 (*n* = 36)
Serum calcium (mg/dL)	9.5 ± 0.5 (*n* = 23)	9.1 ± 1.7 (*n* = 31)	9.5 ± 0.5 (*n* = 39)	8.9 ± 2.3 (*n* = 47)
Serum creatinine (mg/dL)	0.70 ± 0.1 (*n* = 23)	0.96 ± 0.2* (*n* = 31)	0.73 ± 0.1 (*n* = 39)	0.98 ± 0.2* (*n* = 46)
Serum uric acid (mg/dL)	4.4 ± 1.2 (*n* = 23)	6.0 ± 1.2* (*n* = 31)	4.7 ± 1.0 (*n* = 39)	6.4 ± 1.4* (*n* = 47)
Serum phosphate (mg/dL)	3.5 ± 0.5 (*n* = 23)	3.4 ± 0.4 (*n* = 31)	3.7 ± 0.6^†^ (*n* = 39)	3.4 ± 0.6* (*n* = 47)
Urine biochemistry				
pH	6.3 ± 0.5	6.2 ± 0.6	6.2 ± 0.5	6.0 ± 0.6
Osmolality (mOsm)	623 ± 272	664 ± 224	539 ± 238	689 ± 214*
Urine volume (mL/24 h)	1,613 ± 742	1,955 ± 732	1,864 ± 920	1,675 ± 655
Albumin (mg/24 h)	5 ± 13	1.3 ± 3	4 ± 6	6 ± 8^†^
Protein (mg/24 h)	12 ± 16	25 ± 19*	25 ± 16^†^	30 ± 22
Creatinine (mg/24 h)	844 ± 207	1,615 ± 618*	836 ± 302	1,309 ± 628*^†^
Sodium (mmol/24 h)	108 ± 47	180 ± 73*	106 ± 40	146 ± 73*^†^
Potassium (mmol/24 h)	49 ± 27	77 ± 31*	40 ± 21	53 ± 27*^†^
Magnesium (mg/24 h)	108 ± 54	155 ± 81*	89 ± 43	122 ± 63*^†^
Calcium (mg/24 h)	202 ± 81	214 ± 126	175 ± 98	210 ± 124
Oxalate (mg/24 h)	22 ± 8.6	29 ± 15	19 ± 11	24 ± 21
Phosphate (mg/24 h)	645 ± 253	965 ± 468*	581 ± 236	822 ± 414*
Uric acid (mg/24 h)	352 ± 138	599 ± 223*	373 ± 160	447 ± 226^†^
Citrate (mg/24 h)	646 ± 238	637 ± 370	526 ± 281	535 ± 314

Data are presented as mean ± SD and were analyzed by ANOVA followed by Student’s *t* test. *P < 0.05 between men and women of the same group; ^†^ *P* < 0.05 between control and stone formers of the same sex.

The absolute numbers of urinary EVs were normalized to 24-h urine creatinine concentration. Similar results were obtained when urinary EVs were analyzed as a concentration (per microliter of urine) or normalized to urine creatinine (per milligram creatinine).

### Sex differences in populations of urinary EVs between control women and men

The total number of urinary EVs positive for the following markers were significantly (*P* < 0.05) greater in control (non-stone forming) women compared to men: phosphatidylserine (annexin-V), exosome (CD63) marker, inflammatory molecules (VCAM1, tissue factor, and MCP-1), podocin plus galactin-1 (derived from glomerular podocytes), claudin-1 (parietal epithelium of the Bowman’s capsule), urate-anion exchanger (simple cuboidal epithelium of the proximal convoluted tubule), urea transporter (simple squamous epithelium of the thin loop of Henle), uromodulin (simple cuboidal epithelium of the thick loop of Henle), thiazide-sensitive NaCl cotransporter (simple cuboidal epithelium of the distal tubule), aquaporin-2 and V-ATPase (principal and intercalated cells of the collecting duct; principle cell data not shown), cytokeratin 19 (transitional epithelium of the renal pelvis), and neprilysin (urothelium of the urinary bladder) (Tables 1 and 2). There were no differences in numbers of EVs positive for cellular adhesion molecules (E-cadherin and ICAM-1) between control women and men (Table 1).

### Differences in populations of urinary EVs between kidney stone-forming and control women

The total number of exosome (CD63) marker-positive EVs was greater in urine of women with kidney stones compared to control women. However, all of the following EVs were significantly lower (*P* < 0.05) in women with kidney stones: VCAM1-positive EVs and EVs derived from glomerular podocytes, parietal epithelium of the Bowman’s capsule, simple cuboidal epithelium of the proximal convoluted tubule, simple squamous epithelium of the thin loop of Henle, simple cuboidal epithelium of the thick loop of Henle, simple cuboidal epithelium of the distal tubule, principal and intercalated cells of the collecting duct, transitional epithelium of the renal pelvis, and the urothelium of the urinary bladder (Tables 1 and 2). There were no differences in the concentration of E-cadherin, ICAM-1, tissue factor, and MCP-1-positive EVs between women with and without kidney stones (Table 1).

### Sex differences in populations of urinary EVs between stone-forming women and men

Exosome (CD63) marker, inflammatory molecule (MCP-1)-positive EVs, and EVs derived from glomerular podocytes were significantly (*P* < 0.05) lower in stone-forming men compared to stone-forming women (Tables 1 and 2). There were no differences in other population of urinary EVs between stone-forming men and women (Tables 1 and 2).

### Differences in populations of urinary EVs between stone-forming and control men

Total number of phosphatidylserine and exosome (CD63) marker-positive EVs was significantly (*P* < 0.05) greater in stone-forming compared to control men (Table 1). The numbers of urinary EVs positive for other markers did not differ between stone-forming and control men (Tables 1 and 2).

## Discussion

The pathophysiological functions of urinary EVs in kidney stone formation and their cellular origin and numbers in the urine of stone formers and controls have not been established. In this study, we developed and validated methods to quantitate larger (higher than 0.2 μm) urinary EVs from specific nephron segments by digital flow cytometry. Using this standardized technique, we found sex differences in urinary EV populations between men and women who did not have kidney stones and changes in populations of urinary EV in women with and without kidney stones. Women with and without their first episode of symptomatic kidney stones both shed significantly more CD63-positive EVs (indicative of exosomes derived from mature endosome) compared to men, whereas persons of both sexes with renal stones shed a similar number of annexin-V-positive EVs (indicative of microvesicles derived from plasma membrane). Urinary EVs with markers of diverse nephron segments were greater in women without compared to those with kidney stones and men (both with and without stones). The observation that women with kidney stones had urinary EV populations similar to men suggests these markers may reflect renal pathophysiology that causes men as a group to be at a higher risk of kidney stones.

Key challenges are to better understand basic cellular mechanisms involved in kidney stone formation and to identify novel cellular markers for key pathophysiological processes during kidney stone formation. Renal cells can be injured by increased urinary concentrations of lithogenic molecules (e.g., oxalate) or calcium-containing crystals [12]. Activated or injured cells release biologically active EVs (~30 nm to 1,000 nm in diameter; Figure 1). EVs carry biological signatures that reflect their state of differentiation, anatomical location, and function. Growing evidence suggests that EVs in the systemic circulation are involved in numerous pathophysiological processes including cardiovascular disease and cancer, but their role in kidney stone pathophysiology, either in the blood or urine, is not established. EV also participates in the transport of specific cellular signaling molecules from the parent cell to other cells, which in turn alter the biological activity of recipient cells. For example, microvesicles from injured nephrons may promote differentiation of bone marrow stem/precursor cells, inducing them to shed progenitor cells or EVs which might participate in injury processes [18]. However, because the concentration of EVs in the blood and urine depends upon their cell of origin and the stimulus which process triggers their production, populations of urinary EV may reflect an early or late pathophysiological process of kidney stone formation. In other words, they may provide more sensitive and specific markers for the screening, diagnosis, risk stratification, and monitoring of pharmacological therapy for individuals with renal disease, including kidney stones.

Our original hypothesis was that urinary EVs would differ between controls and stone formers in both sexes. Indeed, for most populations of urinary EV, control women formed a unique group and shed a greater amount than either control men or stone-forming women and men. Overall, stone disease is more common in men than women. The current study suggests that the urine EV composition of stone-forming women is similar to that of both control and stone-forming men. Given that control men were similar to stone-forming men, these differences in urine-borne EV populations seem to reflect underlying cellular processes that may correlate with stone risk, rather than a response to ongoing stone formation. However, the underlying reasons for these sex differences in urinary EV populations could be more subtle. It is important to note that the populations of EVs were similar in stone-forming and control men and that the urine of men is in general more supersaturated than women in respect to calcium oxalate. Renal cells can be injured by increased urinary concentrations of oxalate or calcium-containing crystals [12]. Interestingly, the kidney stone precursor lesions called Randall’s plaques are associated with membrane-bound EVs adherent to collagen; these EVs were hypothesized to have induced further growth of more mature plaque along the periphery of the collagen framework [19]. The origin and ultimate fate of these EVs within the papillary interstitium remains to be elucidated. Further characterization of the specific cellular origin, protein, mRNA, micro RNA, and metabolome content of EV in the urine of men and women, with and without kidney stones, is warranted and could identify novel diagnostic and prognostic markers of diseases of the kidney and urinary tract, including kidney stones. Overall, further studies looking at longitudinal cohorts before and after incident stone formation would be necessary to answer this question.

This study clearly highlights the influence of sex on the shedding of urinary EV. The reasons at this point are unclear. It seems likely that hormonal status is important, either by modifying stone risk and urinary crystallization or by direct effects on tubular cell physiology and pathophysiology. Animal studies support this statement as female sex hormones (estrogens) prevent whereas male sex hormones (testosterones) accelerate the progression of kidney stone disease [20,21]. A previous study showed that the total numbers of blood-borne microvesicles were significantly altered by circulating estrogen status in younger healthy postmenopausal women [22]. Our unpublished and other’s published [23] observations also suggest that blood-borne microvesicles positive for phosphatidylserine and platelet antigen are greater in women compared to similar age ranges of apparently healthy men.

Our study has certain limitations. The participants were largely white Americans of European ancestry residing in the upper Midwest. Thus, results will need to be replicated in other ethnic groups and locations. Only first time stone formers were studied, the vast majority of whom (~95%) have calcium oxalate stones. Thus, findings might differ in recurrent stone formers or in other stone types. However, in this study, we have meticulously validated methods to characterize urinary EV and demonstrate for the first time systematic differences between men and women and between stone formers and controls.

## Conclusions

Women in the general population shed a significantly greater number of EVs than men, whereas both stone-forming women and men shed a similar number of most populations of urinary EVs from diverse locations along the urinary tract. These results suggest that women stone formers have urinary EV similar to those in male urine and thus urinary EV shedding may mediate or reflect sex differences in stone pathogenesis. This study also suggests further characterization of the specific cellular origin, protein, mRNA, micro RNA, and metabolome content of EVs in the urine of stone formers and controls is warranted and could identify novel diagnostic and prognostic cellular markers of kidney stones and other diseases of the urinary tract.

## Abbreviations

CD: Cluster of differentiation
EVs: Extracellular vesicles
HEPES: 4-(2-Hydroxyethyl)-1-piperazineethanesulfonic acid
ICAM-1: Inter-cellular adhesion molecule-1
MCP-1: Monocyte chemotactic protein-1
VCAM-1: Vascular cell adhesion molecule-1
